# Favipiravir treatment in non-severe COVID-19: promising results from multicenter propensity score-matched study (FAVICOV)

**DOI:** 10.1038/s41598-023-42195-x

**Published:** 2023-09-09

**Authors:** Taweegrit Siripongboonsitti, Marisa Muadchimkaew, Kriangkrai Tawinprai, Ornisa Issaranon, Wichuda Meepholkij, Pureepat Arttawejkul, Apiradee Vararungzarit, Kaewklao Thavornwattana, Nithi Mahanonda

**Affiliations:** 1 Division of Infectious Diseases, Department of Medicine, Chulabhorn Hospital, Chulabhorn Royal Academy, Bangkok, Thailand; 2 Princess Srisavangavadhana College of Medicine, Chulabhorn Royal Academy, Bangkok, Thailand; 3 Infection Control Unit, Chulabhorn Hospital, Chulabhorn Royal Academy, Bangkok, Thailand; 4 Bang Pa-in Hospital, Phra Nakhon Si Ayutthaya, Thailand; 5 Department of Medicine, Faculty of Medicine, Burapha University Hospital, Chonburi, Thailand; 6 Health Data Science Unit, Department of Medicine, Chulabhorn Hospital, Chulabhorn Royal Academy, Bangkok, Thailand; 7 Chulabhorn Hospital, Chulabhorn Royal Academy, Bangkok, Thailand

**Keywords:** Microbiology, Diseases, Health care, Medical research, Outcomes research

## Abstract

This study aimed to evaluate the efficacy of favipiravir (FPV) in preventing the development of severe COVID-19 in patients with mild-to-moderate symptoms. The study evaluated 1037 COVID-19 patients treated with FPV or standard treatment between April and September 2021, analyzed by propensity score matching. 149 patients were included in each arm after propensity score matching. The clinical outcomes showed no deterioration of the WHO clinical progression scale in the FPV group compared to the standard treatment group on day 5 (83.2% vs. 69.1%, p < 0.001). The WHO clinical progression scale also showed improvements on day 14 in the FPV group compared to the standard treatment group (66.4% vs. 46.3%, p < 0.001). The rates of oxygen supplementation and hospitalization were significantly lower in the FPV group compared to the standard treatment group (0% vs. 12.1% and 0.7% vs. 17.4%, respectively, p < 0.001 for both). There were no differences in adverse events between the two groups. The study highlights the effectiveness of FPV in preventing severe COVID-19 and hospitalization in patients with mild-to-moderate symptoms. The findings emphasize the importance of personalized treatment plans for COVID-19 patients, starting FPV treatment early, and adjusting dosages based on ethnicity and body weight.

## Introduction

The COVID-19 pandemic, stemming from the severe acute respiratory syndrome coronavirus-2 (SARS-CoV-2), has been linked to significant mortality rates. As the evolution of new concerning SARS-CoV-2 variants persists in the aftermath of the pandemic, the dearth of effective anti-SARS-CoV-2 treatments and the challenge of obtaining novel antiviral agents present significant hurdles, particularly in resource-constrained nations.

Novel oral antiviral agents aimed to inhibit viral replication in the early phase to prevent disease progression^1^. Ritonavir-boosted nirmatrelvir (PF-07321332) and molnupiravir stand out as effective pharmaceuticals endorsed for managing mild-to-moderate COVID-19 cases. However, it's important to note that nirmatrelvir presents numerous substantial drug interactions, while the employment of molnupiravir has raised apprehensions regarding potential mutagenic effects in mammalian cells^2,3^.

Favipiravir (FPV) (T-705; 6-fluoro-3-hydroxy-2-pyrazine carboxamide) is an RNA-dependent RNA polymerase (RdRp) inhibitor and purine nucleic acid analog that has demonstrated activity against SARS-CoV-2. Favipiravir is being investigated for COVID-19 treatment but is listed as a drug with a heightened risk of causing significant harm if used incorrectly. Recent studies have shown that FPV treatment is safe and associated with improved clinical and pneumonia symptoms and high virologic clearance^4–7^. FPV treatment has been found to reduce the need for hospitalization, oxygen supplemental, and mechanical ventilation. It has also been shown to shorten the cessation of viral shedding to just 5 days and the time to clinical cure to within 3 days^8,9^. A meta-analysis further supports the benefits of FPV treatment, revealing significant clinical improvements, lower rates of clinical deterioration, greater viral clearance, reduced need for supplemental oxygen therapy, and lower mortality^10,11^.

A recent double-blinded placebo-controlled trial demonstrated the failed efficacy of FPV treatment in mild-to-moderate COVID-19; however, over 80% of patients were the white race, Hispanic, and most comorbidities were hypertension and 76% of overweight and obese^12^. Additionally, the non-linear pharmacokinetics (PK) and auto-inhibition properties of FPV represent essential factors that may impact the interpretation of efficacy and clinical outcomes in overweight and obese patients and specific populations^4,13^. Amidst the surge of the Delta variant, favipiravir was widely used to manage mild to moderate instances of COVID-19. The primary goal was alleviating the strain on healthcare systems grappling with overwhelming caseloads. However, the scarcity of comprehensive data concerning favipiravir's effects within Hispanic populations introduces uncertainties regarding the optimal dosage compared to its administration in Asian populations. Recognizing the variations in treatment approaches across different regions and the importance of tailoring interventions to specific population characteristics, our study evaluates the efficacy of favipiravir treatment. This evaluation encompasses the prevention of severe COVID-19 outcomes and the reduction of hospitalization incidents among patients diagnosed with mild-to-moderate COVID-19.

## Methods

A multicenter, retrospective cohort study was conducted from 1 April 2021 to 30 September 2021 at Chulabhorn Hospital (Bangkok, Thailand) and Bang Pa-In Hospital (Phra Nakhon Sri Ayutthaya, Thailand), respectively. The FPV and standard treatment patients were reviewed from medical records. Some missing information was collected from electronic questionnaires and telephone—the consent to participation, which was waived by the ethics committee. The trial followed the Good Clinical Practice guidelines and the principles of the Declaration of Helsinki. The Human Research, Chulabhorn Research Institute Ethics Committee, approved study no.153/2564. The study was registered in TCTR20221113004.

### Participants

Of 1037 mild-to-moderate COVID-19 patients completing data, 74% of SARS-CoV-2 polymerase chain reaction (PCR)-confirmed and 25.6% of SARS-CoV-2 antigen-confirmed cases collected from an upper respiratory tract specimen that was treated with outpatients, home isolation, or lived in quarantine facilities.

Asymptomatic patients have virologically confirmed infections but no respiratory or gastrointestinal symptoms. Mild COVID-19 is defined as symptomatic cases with at least one respiratory symptom, including fever, cough, myalgia, sore throat, runny nose, dyspnea, chest discomfort, and shortness of breath. Moderate COVID-19 is all pneumonia cases that do not receive supplemental oxygen^14^.

This study excluded patients with age less than 20-year-old, pregnant or breast-feeding conditions, or who lives in long-term facilities or prison, patients who need hospitalization on the initial day, severe or critical COVID-19 respiratory was defined as pneumonia with a respiratory rate > 30 breaths per minute, oxygen saturation ≤ 94% at rest, > 50% pulmonary infiltrates, requiring supplemental oxygenation, respiratory failure, shock, multi-organ failure, COVID-19 severity index > 4, and World Health Organization (WHO) clinical progression scale > 3^1,14–16^.

### Treatment

FPV stands as the primary and most readily accessible antiviral treatment for mild-to-moderate COVID-19 cases, particularly during the outbreaks of the B1.1.7 and B.1.617.2 variants within Thailand. Most patients were sourced from the zenith of a B.1.617.2 variant outbreak, prompting the implementation of widespread FPV treatment. In the FPV-treated cohort, patients were administered an initial loading dose of 3600 mg on the first day, followed by a daily dosage of 1600 mg. Notably, patients with obesity, characterized by a body mass index (BMI) exceeding 35 kg/m^2^, received an initial dose of 4800 mg on the first day, followed by 2000 mg per day for the subsequent four days. These dosages were aligned with the directives outlined in the Thai National COVID-19 Treatment guidelines^17^. In comparison, the standard treatment group was only symptomatic and supportive treatment. All patients in both arms did not receive any potential antiviral activity agents, including FPV, molnupiravir, nirmatrelvir/ ritonavir, azithromycin, and hydroxychloroquine.

### Measurements

The primary outcome evaluated efficacy in preventing the worsening of the WHO clinical progression scale on day 5. The secondary endpoints were also collected, such as improvement of the WHO clinical progression scale on days 5 and 14, oxygen supplemental, and hospitalization from severe COVID-19.

Clinical improvement was defined as two or more symptoms improved by more than 50% after 24 h of starting treatment. Clinical deterioration was defined as one or more symptoms that worsened 24 h after starting treatment. Stable disease was defined as less than two symptoms improved or no change in symptoms.

### Statistical analysis

The sample size was calculated from the 7% and 16% of disease worsening among mild to moderate COVID-19 between FPV and standard treatment from an observational study, respectively. At least 85 participants are needed, leading to the B1.1.7 error of 0.05 and 90% power^7,18^.

Continuous, non-normally distributed data were presented as median (interquartile range [IQR]). Categorical data were analyzed using the Chi-squared test or Fisher's exact test. Non-normally distributed continuous data were compared using the Mann–Whitney *U* test or Student’s t-test between groups.

Matched propensity score, which evaluates confounding by indication and/or baseline covariates between the group, including age, gender, comorbidities, time from onset to treatment, symptomatic, diagnostic methods, vaccinated status, the dose of vaccine received, duration from last vaccination, were analyzed by multivariate logistic regression; the propensity score was used to produce a one-to-one propensity score matching, which was the closest with no replacement. The caliper was 0.2 times the root means square value of the standard deviation of the two groups—a standardized mean difference (SMD) between groups for all covariates. The survival rate was analyzed by log-rank test. The Kaplan–Meier survival curve demonstrated median survival time and compared the survival rate with the log-rank test. Multiple logistic regression analysis was used for multivariate analysis reported with Odds Ratio (OR). A two-sided P value < 0.05 was considered statistically significant, with 90% power and a 95% confidence interval (95%CI). All statistical analyses were performed with Stata version 13.0 (StataCorp, College Station, TX, USA).

### Ethical approval and consent to participate

The Ethics Committee for Human Research, Chulabhorn Research Institute approved the study, no. 153/2564. The Ethics Committee for Human Research, Chulabhorn Research Institute, waived the need for an informed consent requirement.

## Results

### Baseline characteristics

**Figure 1:**
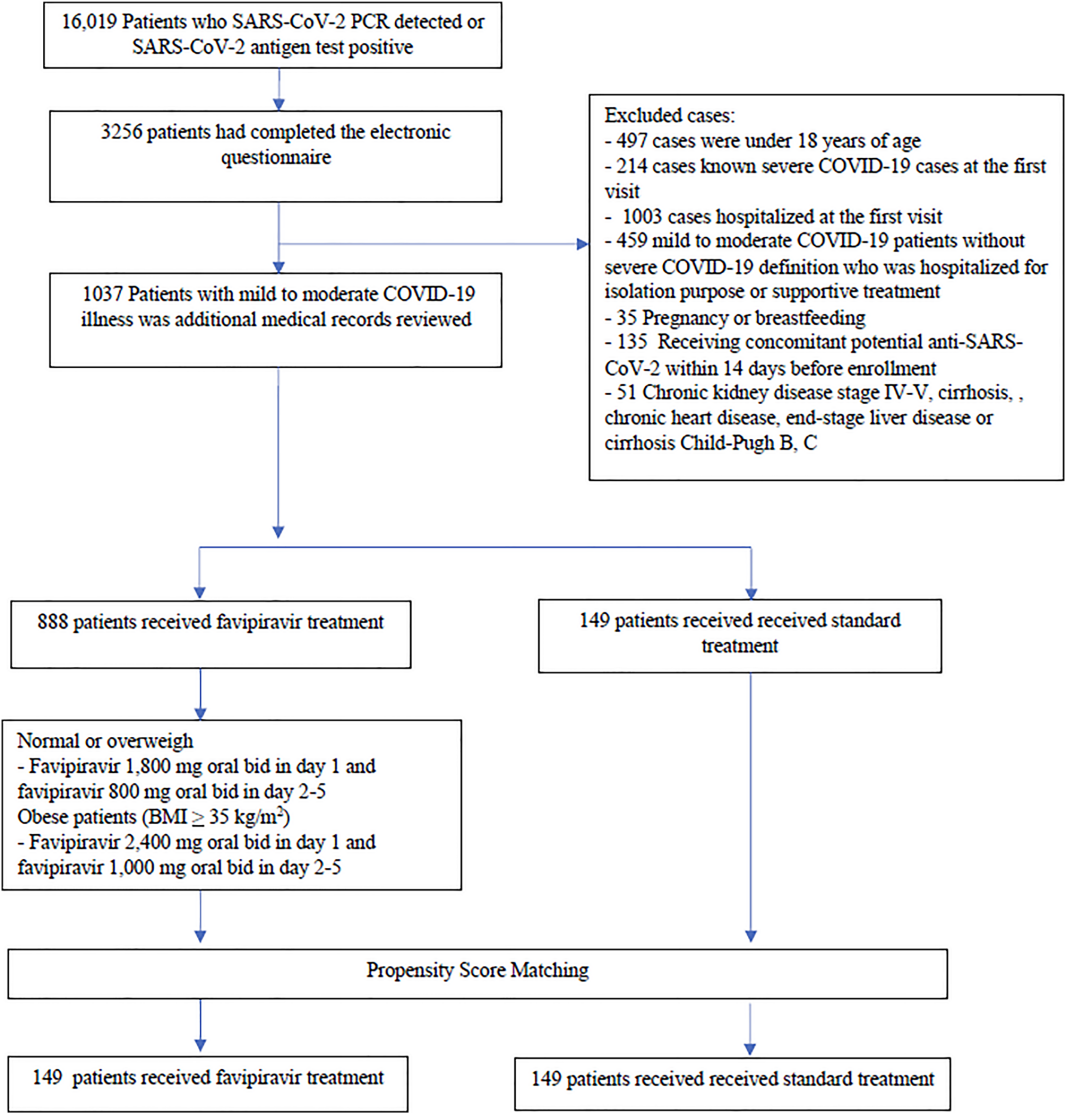
Flow chart of patient selection.

The 888 and 149 patients were eligible for FPV and standard treatments. After baseline covariates matched for propensity score, 149 patients remained in each group (Fig. 1).

Before the propensity score matched, the FPV group had more proportion of some factors than the standard treatment group, including age ≥ 60-year-old (15.5% vs. 2%, p < 0.001), comorbidities (43.4% vs. 24.8%, p < 0.001), hypertension (20% vs. 6.7%, p < 0.001), diabetes mellitus (10.2% vs. 2.7%, p = 0.003), malignancy (3.8% vs. 0.7%, p = 0.048), pneumonia (10.4% vs. 2%, p < 0.001) despite of unknown severity. While the standard treatment group had a higher proportion of unvaccinated patients than the FPV group (Table1).

**Table 1:** Baseline characteristics.

Characteristics	Before propensity score matching (full patient cohort)					After propensity score matching (propensity score matched patient cohort)				
	Total (n = 1037)	Favipiravir (n = 888)	Standard treatment (n = 149)	p-value^a^	SMD	Total (n = 298)	Favipiravir (n = 149)	Standard treatment (n = 149)	p-value^a^	SMD
Age (year-old)	44.11 ± 13.82	44.91 ± 14.13	39.34 ± 10.58	< 0.001	0.447	37.03 ± 10.63	34.73 ± 10.20	39.34 ± 10.58	< 0.001	0.443
< 60	896 (86.4)	750 (84.5)	146 (98.0)	< 0.001	0.492	291 (97.7)	145 (97.3)	146 (98.0)	1.000	0.044
≥ 60	141 (13.6)	138 (15.5)	3 (2.0)			7 (2.3)	4 (2.7)	3 (2.0)		
Male	426 (41.1)	372 (41.9)	54 (36.2)	0.195	0.116	101 (33.9)	47 (31.5)	54 (36.2)	0.392	0.099
Comorbidities	422 (40.7)	385 (43.4)	37 (24.8)	< 0.001	0.398	58 (19.5)	21 (14.1)	37 (24.8)	0.019	0.274
Hypertension	188 (18.1)	178 (20.0)	10 (6.7)	< 0.001	0.399	11 (3.7%)	1 (0.7)	10 (6.7)	0.010	0.326
Cardiovascular disease	27 (2.6)	26 (2.9)	1 (0.7)	0.161	0.170	2 (0.7)	1 (0.7)	1 (0.7)	1.000	0.000
Diabetes mellitus	95 (9.2)	91 (10.2)	4 (2.7)	0.003	0.311	4 (1.3)	0 (0.0)	4 (2.7)	0.122	0.235
Chronic obstructive pulmonary disease	66 (6.4)	57 (6.4)	9 (6.0)	0.861	0.016	21 (7.0)	12 (8.1)	9 (6.0)	0.497	0.079
Obesity (BMI ≥ 30 kg m^−2^)	60 (5.8)	53 (6.0)	7 (4.7)	0.539	0.057	11 (3.7)	4 (2.7)	7 (4.7)	0.357	0.107
Malignancy	35 (3.4)	34 (3.8)	1 (0.7)	0.048	0.214	3 (1.0)	2 (1.3)	1 (0.7)	1.000	0.067
Chronic kidney disease	10 (1.0)	9 (1.0)	1 (0.7)	1.000	0.037	1 (0.3)	0 (0.0)	1 (0.7)	1.000	0.116
Cerebrovascular disease	8 (0.8)	7 (0.8)	1 (0.7)	1.000	0.014	1 (0.3)	0 (0.0)	1 (0.7)	1.000	0.116
HIV infection	6 (0.6)	5 (0.6)	1 (0.7)	1.000	0.014	1 (0.3)	0 (0.0)	1 (0.7)	1.000	0.116
On immunosuppressive agents	7 (0.7)	6 (0.7)	1 (0.7)	1.000	0.001	1 (0.3)	0 (0.0)	1 (0.7)	1.000	0.116
Duration from the onset of illness to treatment										
Asymptomatic	222 (21.4)	183 (20.6)	39 (26.2)	0.581	0.147	80 (26.8)	41 (27.5)	39 (26.2)	0.988	0.066
0–24 h	287 (27.7)	245 (27.6)	42 (28.2)			83 (27.9)	41 (27.5)	42 (28.2)		
24–48 h	286 (27.6)	250 (28.2)	36 (24.2)			71 (23.8)	35 (23.5)	36 (24.2)		
48–72 h	135 (13)	117 (13.2)	18 (12.1)			34 (11.4)	16 (10.7)	18 (12.1)		
≥ 72 h	107 (10.3)	93 (10.5)	14 (9.4)			30 (10.1)	16 (10.7)	14 (9.4)		
Symptoms	990 (95.5)	846 (95.3)	144 (96.6)	0.456	0.070	290 (97.3)	146 (98)	144 (96.6)	0.723	0.083
Fever	675 (65.1)	585 (65.9)	90 (60.4)	0.194	0.114	174 (58.4)	84 (56.4)	90 (60.4)	0.481	0.082
Cough	815 (78.6)	701 (78.9)	114 (76.5)	0.503	0.058	225 (75.5)	111 (74.5)	114 (76.5)	0.686	0.047
Dyspnea	598 (57.7)	514 (57.9)	84 (56.4)	0.788	0.030	164 (55.0)	80 (53.7)	84 (56.4)	0.641	0.054
Sore throat	834 (80.4)	715 (80.5)	119 (79.9)	0.853	0.016	238 (79.9)	119 (79.9)	119 (79.9)	1.000	0.000
Severity										
Mild COVID-19	652 (62.9)	572 (64.4)	80 (53.7)	< 0.001	0.502	147 (49.3)	67 (45.0)	80 (53.7)	0.190	0.212
Moderate COVID-19	95 (9.1)	92 (10.4)	3 (2.0)			10 (3.4)	7 (4.7)	3 (2.0)		
Asymptomatic and unidentified	290 (28)	224 (25.2)	66 (44.3)			141 (47.3)	75 (50.3)	66 (44.3)		
Number of SARS-CoV-2 vaccinated dose										
Unvaccinated	124 (12.0)	97 (10.9)	27 (18.1)	0.012	0.336	67 (22.5)	40 (26.8)	27 (18.1)	0.034	0.380
1 dose	79 (7.6)	67 (7.5)	12 (8.1)			28 (9.4)	16 (10.7)	12 (8.1)		
2 doses	277 (26.7)	238 (26.8)	39 (26.2)			79 (26.5)	40 (26.8)	39 (26.2)		
3 doses	417 (40.2)	355 (40.0)	62 (41.6)			114 (8.3)	52 (34.9)	62 (41.6)		
4 doses	140 (13.5)	131 (14.8)	9 (6.0)			10 (3.4)	1 (0.7)	9 (6.0)		
Homologous booster vaccination	320 (30.9)	268 (30.2)	52 (34.9)	0.015	0.280	105 (35.2)	53 (35.6)	52 (34.9)	0.093	0.297
Heterologous prime boost vaccinated	514 (49.6)	456 (51.4)	58 (38.9)			98 (32.9)	40 (26.8)	58 (38.9)		
The latest type of SARS-CoV-2 vaccinated										
Unvaccinated	124 (12.0)	97 (10.9)	27 (18.1)	0.004	0.389	67 (22.5)	40 (26.8)	27 (18.1)	0.157	0.332
Coronavac	47 (4.5)	43 (4.8)	4 (2.7)			11 (3.7)	7 (4.7)	4 (2.7)		
ChAdOx-1 nCoV	350 (33.8)	304 (34.2)	46 (30.9)			82 (27.5)	36 (24.2)	46 (30.9)		
BBIBP-CorV	56 (5.4)	44 (5.1)	11 (7.4)			22 (7.4)	11 (7.4)	11 (7.4)		
BNT162b2	310 (29.9)	280 (31.5)	30 (20.1)			66 (22.1)	36 (24.2)	30 (20.1)		
mRNA-1273	149 (14.4)	118 (13.3)	31 (20.8)			50 (16.8)	19 (12.8)	31 (20.8)		
Ad26.COV2.S	1 (0.1)	1 (0.1)	0 (0.0)			0 (0)	0 (0)	0 (0)		
Duration after the last COVID-19 vaccination										
Unvaccinated	124 (12)	97 (10.9)	27 (18.1)	0.034	0.249	67 (22.5)	40 (26.8)	27 (18.1)	0.230	0.242
< 14 days	84 (8.1)	69 (7.8)	15 (10.1)			31 (10.4)	16 (10.7)	15 (10.1)		
14–90 days	563 (54.3)	486 (54.7)	77 (51.7)			149 (50.0)	72 (48.3)	77 (51.7)		
> 90 days	266 (25.7)	236 (26.6)	30 (20.1)			51 (17.1)	21 (14.1)	30 (20.1)		
SARS-COV-2 diagnostic method										
SARS-CoV-2 PCR	772 (74.4)	655 (73.8)	117 (78.5)	0.217	0.112	236 (79.2)	119 (79.9)	117 (78.5)	0.775	0.033
SARS-CoV-2 antigen	265 (25.6)	233 (26.2)	32 (21.5)			62 (20.8)	30 (20.1)	32 (21.5)		

*NA* data not applicable, *PCR* polymerase chain reaction, *SARS-CoV-2* severe acute respiratory syndrome coronavirus-2, *COVID-19* coronavirus disease-2019, *n* number.

Data are presented as number (%), mean ± standard deviation, or median (interquartile range).

^a^P-value corresponds to the independent samples t-test, Mann–Whitney *U* test, Chi-square test, or Fisher’s exact test.

One-to-one propensity score matching was also performed. Logistic regression was used to calculate a propensity score, which evaluates confounding by indication and/ or baseline covariates between two insurance groups.

A standardized mean difference (SMD) between groups for all covariates is shown in Table 1.

After the propensity score was matched, most baseline characteristics were not different. However, the standard treatment had older patients than the FPV group (p < 0.001). In contrast, 26.8% and 18.1% of patients were unvaccinated in FPV and standard treatment groups. The third and fourth dose booster vaccination predominated in the standard treatment (p = 0.034). No discernible distinctions were observed in variables such as age exceeding 60 years, high-risk comorbidities, the duration from symptom onset to treatment initiation, COVID-19 vaccination status, specific vaccine types, symptomatic cases, and pneumonia incidence. Notably, the temporal interval between the onset of illness and the commencement of treatment was limited to 72 h for 90% of individuals within both cohorts.

### Treatment results

The primary outcome analysis revealed that the FPV arm and the standard treatment arm exhibited an absence of clinical deterioration on the WHO clinical progression scale on day 5 at rates of 86.5% and 69.1%, respectively (p < 0.001, adjusted odds ratio [aOR] 4.92, 95% confidence interval [CI] [2.64, 9.20]), prior to propensity score matching. After propensity score matching, these figures were 83.2% and 69.1% (p < 0.001, aOR 3.48, 95% CI [1.68, 7.20]). (Table 2).

**Table 2:** Outcomes of favipiravir and standard treatment demonstrated by propensity score matching.

Outcome	Before propensity score matching (full patient cohort)								After propensity score matching (propensity score matched patient cohort)								
	FPV (n = 888)	Control (n = 149)	Estimated difference (95% CI)	p-value^a^	Odds ratio (95% CI)	p-value^b^	Adjusted odds ratio (95% CI)	p-value^b^		FPV (n = 149)	Control (n = 149)	Estimated difference (95% CI)	p-value^a^	Odds ratio (95% CI)	p-value^b^	Adjusted odds ratio (95% CI)	p-value^b^
WHO clinical progression scale, not deterioration																	
Day 5	768 (86.5)	103 (69.1)	17.4 (9.8, 25.3)	< 0.001	2.86 (1.92, 4.25)	< 0.001	4.92 (2.64, 9.20)	< 0.001		124 (83.2)	103 (69.1)	14.1 (4.4, 23.4)	0.004	2.22 (1.28, 3.85)	0.005	3.48 (1.68, 7.20)	< 0.001
Day 14	874 (98.4)	145 (97.3)	1.1 (− 1.4, 4.6)	0.312	1.72 (0.56, 5.31)	0.344	1.34 (0.34, 5.22)	0.673		145 (97.3)	145 (97.3)	0 (− 4.0, 4.0)	1.000	1.00 (0.25, 4.08)	1.000	1.43 (0.27, 7.54)	0.673
Improved WHO clinical progression scale																	
Day 5	151 (17.0)	18 (12.1)	4.9 (− 1.3, 10.3)	0.132	1.49 (0.88, 2.52)	0.134	1.23 (0.70, 2.16)	0.480		23 (15.4)	18 (12.1)	3.4 (− 4.6, 11.2)	0.400	1.33 (0.68, 2.58)	0.401	1.63 (0.75, 3.54)	0.220
Day 14	654 (73.6)	69 (46.3)	27.3 (18.8, 35.7)	< 0.001	3.24 (2.27, 4.62)	< 0.001	7.49 (4.43, 12.67)	< 0.001		99 (66.4)	69 (46.3)	20.1 (8.9, 30.8)	< 0.001	2.30 (1.44, 3.67)	< 0.001	9.74 (3.98, 23.79)	< 0.001
Clinical improvement																	
Day 5	779 (87.7)	105 (70.5)	17.3 (9.8, 25.1)	< 0.001	3.00 (2.00, 4.49)	< 0.001	3.20 (2.05, 4.99)	< 0.001		133 (89.3)	105 (70.5)	18.8 (9.7, 27.4)	< 0.001	3.48 (1.86, 6.52)	< 0.001	4.03 (2.01, 8.07)	< 0.001
Day 14	793 (89.3)	122 (81.9)	7.4 (1.2, 14.3)	0.009	1.85 (1.16, 2.95)	0.010	1.69 (1.03, 2.77)	0.039		130 (87.2)	122 (81.9)	5.4 (− 2.9, 13.5)	0.200	1.51 (0.80, 2.86)	< 0.001	1.77 (0.88, 3.56)	0.109
Oxygen supplemental	6 (0.7)	18 (12.1)	− 11.4 (− 17.1, − 6.5)	< 0.001	0.05 (0.02, 0.13)	< 0.001	0.02 (0.01, 0.07)	< 0.001		0 (0.0)	18 (12.1)	− 12.1 (− 17.4, − 6.5)	< 0.001	–	NA	–	NA
Hospitalization	10 (1.1)	26 (17.4)	− 16.3 (− 23.2, − 11)	< 0.001	0.05 (0.03–0.11)	< 0.001	0.02 (0.01, 0.07)	< 0.001		1 (0.7)	26 (17.4)	− 16.8 (− 22.9, − 10.2)	< 0.001	0.03 (0.01, 0.24)	< 0.001	0.02 (0.01, 0.14)	< 0.001

*95% CI* 95% Confidence interval, *NA* data not applicable, *n* number, *WHO* World Health Organization.

Data are presented as numbers (%).

^a^Estimated difference is the difference in proportions shown for favipiravir compared to the control.

^b^Effect estimate is the odds ratio (2-sided 95% CI) from a logistic model.

Furthermore, the secondary outcome analysis demonstrated that the improvement in the WHO clinical progression scale on day 14 was more pronounced in the FPV and standard treatment groups, with rates of 66.4% and 46.3%, respectively (p < 0.001, aOR 9.74, 95% CI [3.98, 23.79]). Nevertheless, the differentiation between both groups regarding improving the WHO clinical progression scale on day 5 and the absence of clinical deterioration on day 14 was not statistically significant (p = 0.220, p = 0.673).

Before the propensity score matched, overall clinical improvement was 87.7%, 89.3% in the FPV and 70.5%, 81.9% in the standard treatment group on day 5 (p < 0.001, aOR 1.69, p < 0.001; 95% CI [2.05, 4.99]), and day 14, respectively (p = 0.009, aOR 3.20, p = 0.039; 95% CI [1.03, 2.77]). After the application of propensity score matching, the clinical enhancement figures on day 5 were 89.3% and 70.5% for the FPV and standard treatment groups, respectively, along with rates of 87.2% and 81.9% on day 14 (p < 0.001, aOR 4.03, p < 0.001, 95% CI [2.01, 8.07]; p = 0.200, aOR 1.77, p = 0.109, 95% CI [0.88, 3.56]).

However, no difference was observed in VAS score improvement of respiratory symptoms between the FPV and standard treatment groups on day 0 and day 5. No difference in time to any symptom’s resolution and many symptomatic improved patients were observed in both groups. The time to resolution of overall symptoms was 6 IQR (4, 9) days and 6 IQR (4, 8) days in FPV and standard treatment groups, respectively (p = 0.809; aHR 0.97, 95% CI [0.74, 1.26]) (Supplementary Tables 1–3).

**Figure 2:**
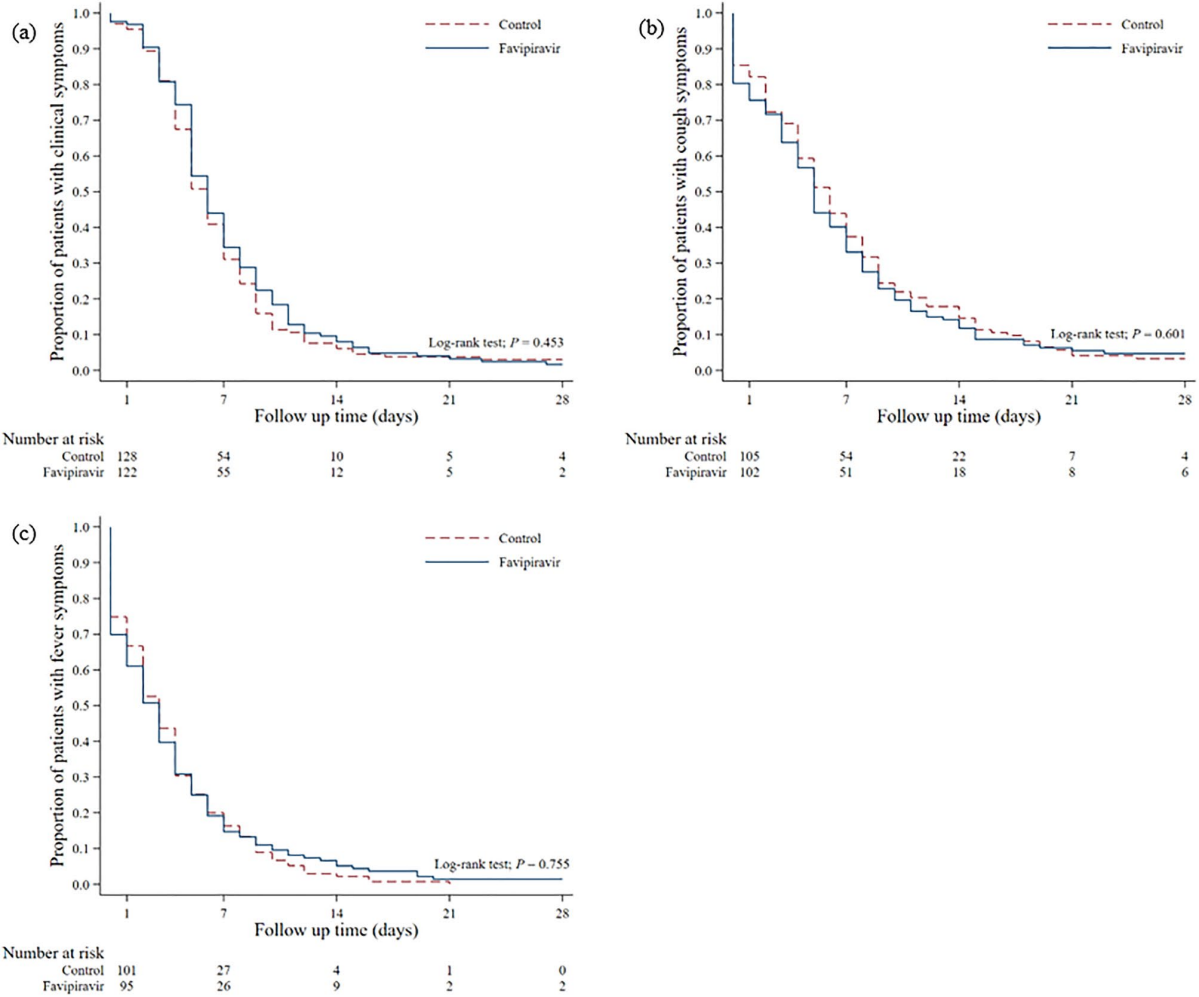
Kaplan–Meier curve of the proportion of patients with (**a**) overall clinical symptoms, (**b**) cough, (**c**) fever.

The Kaplan–Meier curve illustrated that the median duration of persistent respiratory symptoms was consistent at six days for both the FPV and standard treatment groups (p = 0.453). Furthermore, the median duration of lingering fever and coughing was observed to be 3 and 5 days, respectively, in the FPV group, while in the standard treatment group, these durations were 3 and 6 days (p = 0.755, p = 0.601) (Fig. 2, Supplementary Table 4).

A greater proportion of patients necessitated oxygen supplementation in the standard treatment group compared to the FPV group, with rates of 12.1% and 0.7%, respectively, before propensity score matching (p < 0.001; aOR 0.02, 95% CI [0.01, 0.07]). The hospitalization rate within the standard treatment group was notably higher at 17.4%, in contrast to the 1.1% observed in the FPV group (p < 0.001). Following propensity score matching, the discrepancy remained significant, with the standard treatment group showing a higher requirement for oxygen supplementation (0% vs. 12.1%, p < 0.001), as well as a higher hospitalization rate (0.7% vs. 17.4%, p < 0.001; aOR 0.2, 95% CI [0.01, 0.14]) (Table 2).

### Adverse events

No difference in adverse events and severity of adverse events was observed in both groups after propensity score matching (Table 3, Supplementary Table 5).

**Table 3:** Incidence of adverse events evaluated by propensity score matching.

Adverse events	Before propensity score matching (full patient cohort)				After propensity score matching (propensity score matched patient cohort)			
	Favipiravir (n = 888)	Standard treatment (n = 149)	Estimated difference (95% CI)^a^	p-value^b^	Favipiravir (n = 149)	Standard treatment (n = 149)	Estimated difference (95% CI)^a^	p-value^b^
Nausea	71 (8.0)	20 (13.4)	− 5.4 (− 12 to − 0.5)	0.030	16 (10.7)	20 (13.4)	− 2.7 (− 10.1 to 4.8)	0.477
Vomiting	27 (3.0)	11 (7.4)	− 4.3 (− 9.8 to − 0.9)	0.009	7 (4.7)	11 (7.4)	− 2.7 (− 8.3 to 3.0)	0.331
Diarrhea	122 (13.7)	20 (13.4)	0.3 (− 6.4 to 5.5)	0.917	24 (16.1)	20 (13.4)	2.7 (− 5.5 to 10.7)	0.514
Eye discoloration	23 (2.6)	5 (3.4)	− 0.8 (− 5.1 to 1.5)	0.584	3 (2.0)	5 (3.4)	− 1.3 (− 5.4 to 2.7)	0.474
Jaundice	9 (1.0)	3 (2.0)	− 1.0 (− 4.8 to 0.6)	0.396	0 (0.0)	3 (2.0)	− 2.0 (− 4.9 to 0.9)	0.082
Arthralgia	52 (5.9)	9 (6.0)	− 0.2 (− 5.4 to 3.1)	0.929	11 (7.4)	9 (6.0)	1.3 (− 4.5 to 7.2)	0.643
Palpitation and sweating	67 (7.5)	14 (9.4)	− 1.9 (− 7.8 to 2.3)	0.436	10 (6.7)	14 (9.4)	− 2.7 (− 9.0 to 3.7)	0.394
Abdominal pain	14 (1.6)	2 (1.3)	0.2 (− 3.2 to 1.7)	1.000	0 (0.0)	2 (1.3)	− 1.3 (− 3.9 to 1.2)	0.156
Dizziness	84 (9.5)	19 (12.8)	− 3.3 (− 9.8 to 1.6)	0.214	13 (8.7)	19 (12.8)	-4.0 (− 11.1 to 3.1)	0.262
Difficult breathing	37 (4.2)	9 (6.0)	− 1.9 (− 7.0 to 1.3)	0.304	4 (2.7)	9 (6.0)	− 3.4 (− 8.2 to 1.6)	0.156
Facial edema	8 (0.9)	1 (0.7)	0.2 (− 2.8 to 1.3)	1.000	2 (1.3)	1 (0.7)	0.7 (− 2.2 to 3.5)	0.562
Allergic rash	39 (4.4)	7 (4.7)	− 0.3 (− 5.1 to 2.6)	0.867	8 (5.4)	7 (4.7)	0.7 (− 4.5 to 5.9)	0.791

*NA* data not applicable; coronavirus disease-2019, *n* number.

Data are presented as numbers (%).

^a^An estimated difference is the difference in proportions shown for favipiravir compared to the control.

^b^p-value corresponds to the Chi-square test or Fisher’s exact test.

## Discussion

This multicenter study presents a valuable perspective on the effectiveness of FPV treatment within real-world contexts, specifically focusing on individuals with mild to moderate COVID-19 undergoing either home isolation or quarantine in designated facilities. Notably, the investigation was conducted during the peak of the SARS-CoV-2 pandemic in Thailand, marked by the prevalence of the B.1.617.2 variant outbreak. The study's outcomes are particularly significant, revealing that patients receiving FPV experienced a significantly lower incidence of clinical deterioration leading to severe COVID-19, as measured by the WHO clinical progression scale on day 5. This observation held true both prior to and following propensity score matching.

Moreover, the FPV-treated group exhibited a greater proportion of patients demonstrating clinical improvement than the standard treatment group on day 14. These findings emphasize the potential efficacy of FPV as a promising therapeutic strategy for COVID-19 patients, particularly within real-world scenarios where the urgent need for effective treatment options remains a critical concern.

Our results were consistent with the recent report that the FPV-treated group prevented deterioration to severe disease in 79.1% of the patients, 76.9% of moderate cases, and 86.2% of mild cases on day 7^7^. The recent study could not demonstrate that early and late FPV treatment had a difference in viral clearance on day 6 in FPV and control groups, although times to defervescence was shorter, 2.1 days in early FPV treatment compared to and 3.2 days in late treatment groups (aOR 1.88; 95% CI 0.81–4.35)^19^.

A randomized control trial (RCT) in Thailand, which had a mean duration from symptoms to FPV treatment of 1.6 days, showed clinical improvement within 14 days, including 79% in FPV and 32.3% in the control group (p < 0.001). The majority were middle-aged, mild COVID-19, and consisted of D614G ancestral strain (26.8%), B1.1.7 (66.1%), and B.1.617.2 (7.1%)^20^. It corresponded with our results that showed significant clinical improvement of 73.6%, 66.4% in FPV, and 46.3%, 46.3% in the control group before and after propensity score matching on the WHO clinical progression scale at 14 days. It demonstrated preventing disease progression 86.5%, 83.2%, in FPV and 69.1%, 69.1% in standard treatment groups before and after propensity score matching on the WHO clinical progression scale on day 5, although there were more unvaccinated patients in the FPV-treated group. The data complied with a meta-analysis that revealed significant clinical improvement after 7 days and within 14 days^10,21^. The critical issue in our study was that 89.7% of patients received early FPV within 72 h after symptoms onset, corresponding to results from a previous study that showed a positive correlation between the duration of fever defervescence and early time to initiate FPV treatment^22^.

Many RCTs showed the controversial efficacy of FPV. A study used the fixed-dose 1800 mg FPV twice daily as a loading dose on day 1, followed by 800 mg twice daily as a maintenance dose for 5 to 7 days in all body weight. The study in mild COVID-19 patients showed indifferent time to viral clearance, time to recovery, hospitalization, and ICU admission. However, the FPV-treated group had 21.4% BMI ≥ 30 kg/m^2^ without an increasing dose of FPV^23^. In contrast to our study, there was 5.6% of patients with BMI ≥ 30 kg/m^2^.

The open-label RCT in Malaysia showed FPV did not prevent disease progression to severe COVID-19 and 18% vs. 14.8% of severe disease developed in FPV and control, respectively; however, the day of illness to FPV was 5 days in both groups, 16.4% required oxygen supplemental at baseline, many other agent contaminations and interpreted as entirely delay to take the antiviral drug, 20% of obese patients in both groups^24^. A small RCT showed no clinical benefits of FPV in symptomatic mild to moderate COVID-19. However, this study did not clearly describe many essential points, including baseline characteristics, especially comorbidities, pulmonary involvement, vaccination status, and symptoms in the last 5 days, while the majority of the WHO clinical progression scale equal to 2^25^.

PRESECO study demonstrated a lack of efficacy of early FPV treatment in mild-to-moderate COVID-19 represented by time to sustained clinical recovery in 80% of Hispanic, white race, and one-fourth demonstrated seropositive patients, and 76% of overweight (BMI ≥ 25 kg/m^2^). However, based on a complex non-linear pharmacokinetic profile, the dose of FPV in the study might be quite low, especially in obese patients, resulting in suboptimal therapeutic concentration^12^.

A recent study in COVID-19 with 46.5% SARS-CoV-2 PCR confirmed cases found that 18.1% required supplemental oxygen in chest CT detected pneumonia in FPV treated^5^. The hospitalization rate was 5.3% in FPV-treated mild COVID-19^23^. The study in high-risk mild to moderate COVID-19 found that 18.4% required oxygen supplemental and 2.4% required mechanical ventilation in the FPV-treated group^24^. Moreover, the meta-analysis demonstrated that the FPV group required oxygen supplemental 7% less than the control group^10^. In our study, the less mild to moderate COVID-19 patients who required hospitalization and oxygen supplemental might result from earlier loading and maintenance dose-weight adjusted FPV treatment.

FPV treatment outcomes had interpersonal variability despite well-designed studies. Based on PK data, the FPV trough concentration substantially decreased by 89% between day 2 (21.26 μg/mL) and day 4 (1.6 μg/mL) after COVID-19 treatment in Turkish, with the majority having BMI > 25 kg/m^2^ and predicted to decrease over time, while, the plasma concentration need > 20 μg/mL to achieve the 50% effective concentration (EC50) value (9.4 μg/mL). However, the trough concentration in healthy volunteers ranged from 20 to 60 μg/mL (Day 1: 600 mg bid, day 2–5: 600 mg bid)^26–28^. The population PK study showed that body surface area, dosage, and invasive mechanical ventilation were related to clearance and bioavailability. The study showed that a double dose regimen (3200/ 1200 b.i.d.) was lower trough concentration than EC50, and 1600 mg b.i.d. was the only regimen exceeding EC50 in a patient with BSA = 1.72 but not BSA = 2.2. The sufficient maintenance dose of FPV should be between 800 and 1200 mg twice daily and could provide the therapeutic concentration of active intracellular FPV metabolite^29^.

Our study implied that the potential clinical benefits of FPV treatment would occur under conditions including appropriate loading and maintenance dosage of FPV to body weight and starting early in the course < 72 h after the onset of illness. The Asian ethnicity might be the main factor in predicting the favorable outcome of FPV to mild-to-moderate COVID-19 treatment above Americans and Caucasians, which had a 50% plasma concentration of Japanese^30^.

FPV stands out as an early treatment option for individuals of Asian descent who present with mild to moderate COVID-19. Moreover, it is crucial to carefully consider appropriate dosage adjustments for FPV, particularly in the case of obese patients. Throughout the outbreak, FPV has consistently been favored as the treatment choice for mass treatment, especially in regions where alternative antiviral options with robust support from well-designed RCTs are lacking, such as resource-limited countries or some situations. FPV may also be preferred for individuals experiencing significant drug interactions with nirmatrelvir/ritonavir or expressing concerns about potential mutagenesis in mammalian cells. Furthermore, for countries facing continuous outbreaks of new variants of concern and limited resources, FPV holds promise as a viable treatment option. This study emphasizes the importance of widespread FPV treatment to manage mild-to-moderate COVID-19 cases during the current outbreak, underscoring its potential to alleviate the strain on the healthcare system.

There were several limitations in our study. Firstly, the study is retrospective and has the potential to recall bias and other types of error. In addition, the study relied on self-assessment by questionnaires, which can also introduce bias and inaccuracies in the data. Secondly, the study only included patients who could communicate their symptoms and did not include those who had critical COVID-19, died or had communication problems. Thus, the study may not represent the full spectrum of COVID-19 severity. Third, despite our comprehensive analysis of the data using a 14-day deterioration endpoint and the patients' self-reported time to recovery, our study still has certain limitations in assessing those who experienced deterioration after the initial 14-day period. Fourth, our study primarily focused on evaluating the effectiveness of FPV during the extensive administration of FPV treatment at the peak of the B1.1.7 and B.1.617.2 outbreaks. Consequently, the generalizability of our findings to other variants may be restricted. Fifth, only 40% of high-risk and 5.8% of obese patients were included in the study, and the exact BMI of the patients was not reported. Despite variations in baseline characteristics such as malignancy, chronic kidney disease, cerebrovascular disease, HIV infection, and receiving of immunosuppressive agents, no patients in the FPV group could be successfully matched using propensity scores. In addition, 4.5% of patients were asymptomatic. This could limit the generalizability of the study's findings to a broader population of patients with COVID-19. Sixth, the standard treatment group had a higher proportion of unvaccinated patients than the FPV group before propensity score matching, but the proportion of unvaccinated patients was reversed after matching, which might be from confounding factors.

## Conclusion

The real-world data analysis has uncovered compelling evidence of the clinical benefits of early administration of FPV in mild to moderate cases of COVID-19, administered within 72 h of the onset of symptoms. This treatment has shown promise in preventing the development of severe COVID-19, which requires hospitalization or oxygen support. The beneficial effects of this treatment have been observed primarily in Asian patients with low to average body weight. The randomized control trial under appropriate FPV dosage in obese patients and PK study, or increased loading and maintenance dose for achieving trough concentration balancing with toxicities, is challenged and needs further study.

### Supplementary Information

Supplementary Tables.

## Data Availability

The datasets generated and analyzed during the current study are not publicly available due to avoiding use for wrong purposes but are available from the corresponding author on reasonable request.
