# Supplementary material for: Favipiravir treatment in non-severe COVID-19: promising results from multicenter propensity score-matched study (FAVICOV)
Source: Sci Rep. 2023 Sep 9;13:14884. doi: 10.1038/s41598-023-42195-x (PMC10492810; doi:10.1038/s41598-023-42195-x)
Supplement: Supplementary file 1 — Supplementary Tables. [file 41598_2023_42195_MOESM1_ESM.pdf]

Supplementary Material

Contents

Tables

Table1: The propensity score matching of respiratory symptoms and visual analog score between favipiravir and standard treatment groups ……………………………………………………………………….…………….…………….....1

Table2: Time to resolution of symptoms among favipiravir and standard treatment analyzed by propensity score matching ……………………………………………….………………………………………………….…....…2

Table3 Resolution of respiratory tract infection symptoms from COVID-19 on day 5 of favipiravir and standard treatment ……...……………………………………………………………………………………........3

Table4: The residual symptoms on day five after favipiravir and standard treatment ………………….………………4

Table5: Severity of adverse events in favipiravir and standard treatment 9……………………....…………….………………5

Supplementary

Table 1 The propensity score matching of respiratory symptoms and visual analog score between favipiravir and standard treatment groups

Symptoms	Before Propensity Score Matching (Full Patient Cohort)				After Propensity Score Matching (Propensity Score Matched Patient Cohort)			
	**Favipiravir (n = 888)**	**Standard treatment (n = 149)**	**Estimated difference (95% CI)[a]**	**p-valu[b]**	**Favipiravir (n = 149)**	**Standard treatment (n = 149)**	**Estimated difference (95% CI)[a]**	**p-value[b]**
Overall symptoms								
Day 0	846 (95.3)	144 (96.6)	-1.4 (-3.9 to 3.1)	0.456	146 (98.0)	144 (96.6)	1.3 (-2.7 to 5.4)	0.474
Day 5	751 (84.6)	122 (81.9)	2.7 (-3.2 to 10.0)	0.404	120 (80.5)	122 (81.9)	-1.3 (-10.2 to 7.6)	0.767
Fever								
Day 0	585 (65.9)	90 (60.4)	5.5 (-2.7 to 14.1)	0.194	84 (56.4)	90 (60.4)	-4 (-15.1 to 7.1)	0.481
VAS, median (IQR)	2 (0 - 6)	2 (0 - 5.5)	0.29 (-0.31 to 0.88)	0.284	1 (0 - 6.5)	2 (0 - 5.5)	-0.07 (-0.86 to 0.72)	0.688
Day 5	338 (38.1)	50 (33.6)	4.5 (-4.0 to 12.3)	0.293	55 (36.9)	50 (33.6)	3.4 (-7.5 to 14.1)	0.544
VAS, median (IQR)	0 (0 - 2)	0 (0 - 2)	0.18 (-0.23 to 0.60)	0.369	0 (0 - 2)	0 (0 - 2)	0.18 (-0.34 to 0.7)	0.555
Cough								
Day 0	701 (78.9)	114 (76.5)	2.4 (-4.3 to 10.3)	0.503	111 (74.5)	114 (76.5)	-2 (-11.7 to 7.8)	0.686
VAS, median (IQR)	3 (1 - 7)	3 (1 - 6)	0.09 (-0.49 to 0.67)	0.748	2 (0 - 6)	3 (1 - 6)	-0.31 (-1.08 to 0.46)	0.327
Day 5	606 (68.2)	104 (69.8)	-1.6 (-9.0 to 6.8)	0.705	91 (61.1)	104 (69.8)	-8.7 (-19.3 to 2.1)	0.113
VAS, median (IQR)	2 (0 - 5)	2 (0 - 6)	-0.33 (-0.86 to 0.21)	0.304	1 (0 - 5)	2 (0 - 6)	-0.49 (-1.22 to 0.24)	0.122
Dyspnea								
Day 0	514 (57.9)	84 (56.4)	1.5 (-6.9 to 10.1)	0.730	80 (53.7)	84 (56.4)	-2.7 (-13.9 to 8.6)	0.641
VAS, median (IQR)	1 (0 - 5)	1 (0 - 5)	0.04 (-0.51 to 0.59)	0.792	1 (0 - 4.5)	1 (0 - 5)	-0.29 (-1.01 to 0.43)	0.472
Day 5	492 (55.4)	85 (57.0)	-1.6 (-10.0 to 7.0)	0.709	69 (46.3)	85 (57.0)	-10.7 (-21.8 to 0.6)	0.064
VAS, median (IQR)	1 (0 - 4)	1 (0 - 5)	-0.22 (-0.74 to 0.30)	0.427	0 (0 - 3.5)	1 (0 - 5)	-0.56 (-1.23 to 0.11)	0.062
Sore throat								
Day 0	715 (80.5)	119 (79.9)	0.7 (-5.6 to 8.2)	0.853	119 (79.9)	119 (79.9)	0 (-9.1 to 9.1)	1.000
VAS, median (IQR)	4 (1 - 8)	4 (1 - 8)	-0.22 (-0.83 to 0.39)	0.508	3 (1 - 7)	4 (1 - 8)	-0.52 (-1.31 to 0.28)	0.249
Day 5	525 (59.1)	94 (63.1)	-4.0 (-12.0 to 4.6)	0.361	85 (57.0)	94 (63.1)	-6 (-17.0 to 5.1)	0.287
VAS, median (IQR)	1 (0 - 4)	2 (0 - 5)	-0.47 (-0.95 to 0.02)	0.097	1 (0 - 4)	2 (0 - 5)	-0.44 (-1.11 to 0.23)	0.183

Abbreviations: n, number; IQR, interquartile range; 95%CI, 95% confidence interval

a An estimated difference is the proportion difference shown for favipiravir compared to the control.

b P-value corresponds to the Chi-square test or Fisher’s exact test.

Supplementary

Table 2 Time to resolution of symptoms among favipiravir and standard treatment analyzed by propensity score matching

Outcomes	Before Propensity Score Matching (Full Patient Cohort)							After Propensity Score Matching (Propensity Score Matched Patient Cohort)						
	**Favipiravir (n = 888)**	**Control (n = 149)**	**p-value**	**Hazard Ratio (95% CI) [b]**	**p-value**	**Adjusted Hazard ratio (95% CI)**	**p-value**	**Favipiravir (n = 149)**	**Control (n = 149)**	**p-value**	**Hazard Ratio (95% CI) [b]**	**p-value**	**Adjusted Hazard Ratio (95% CI)**	**p-value**
Time, resolution of symptoms														
Overall symptoms (days), median (IQR)	6 (4 - 9)	6 (4 - 8)	0.353	0.93	0.487	0.94	0.527	6 (4 - 9)	6 (4 - 8)	0.453	0.92	0.495	0.97	0.809
				(0.77, 1.13)		(0.77, 1.14)					(0.72, 1.18)		(0.74, 1.26)	
Fever (days), median (IQR)	3 (0 - 5)	3 (0 - 6)	0.385	0.93	0.438	1.00	0.999	3 (0 - 5)	3 (0 - 6)	0.755	0.97	0.781	1.02	0.904
				(0.77, 1.12)		(0.83, 1.21)					(0.76, 1.23)		(0.78, 1.32)	
Cough (days), median (IQR)	5 (3 - 10)	6 (2 - 9)	0.996	1.00	0.996	1.09	0.405	5 (2 - 9)	6 (2 - 9)	0.601	1.06	0.626	1.2	0.206
				(0.82, 1.21)		(0.89, 1.34)					(0.83, 1.37)		(0.90, 1.60)	
Sore throat (days), median (IQR)	4 (0 - 11)	4 (0 - 9)	0.418	0.93	0.459	1.07	0.543	3 (0 - 8)	4 (0 - 9)	0.244	1.14	0.294	1.31	0.054
				(0.77, 1.13)		(0.87, 1.31)					(0.89, 1.47)		(1.00, 1.72)	

Abbreviations: n, number; IQR, interquartile range; 95%CI, 95% confidence interval

a The P-value for this ratio was calculated with the log-rank test.

b Effect estimate is the Hazard ratio for resolution of symptoms was estimated by the Cox proportional-hazard model.

Supplementary

Table 3 Resolution of respiratory tract infection symptoms from COVID-19 on day 5 of favipiravir and standard treatment

Outcome	Favipiravir (n = 888)	Standard treatment (n = 149)	Estimated difference (95% CI)[b]	P-value	Odds ratio (95% CI)	P-value[c]	Adjusted Odds ratio (95% CI)	P-value[c]
Overall symptoms; n (%)	137 (15.4)	27 (18.1)	-2.7	0.404	0.82	0.405	0.79	0.342
			(-10.0to 3.2)		(0.52, 1.30)		(0.48, 1.29)	
Fever; n (%)	550 (61.9)	99 (66.4)	-4.5	0.293	0.82	0.293	0.84	0.416
			(-12.3, 4.0)		(0.57, 1.19)		(0.56, 1.27)	
Cough; n (%)	282 (31.8)	45 (30.2)	1.6	0.705	1.08	0.705	1.16	0.490
			(-6.8, 9.0)		(0.74, 1.57)		(0.76, 1.79)	
Dyspnea; n (%)	396 (44.6)	64 (43.0)	1.6	0.709	1.07	0.709	1.34	0.247
			(-7.0, 10.0)		(0.75, 1.52)		(0.82, 2.19)	
Sore throat; n (%)	363 (40.9)	55 (36.9)	4.0	0.361	1.18	0.362	1.25	0.306
			(-4.6, 12.0)		(0.83, 1.69)		(0.82, 1.9)	

Abbreviations: n, number; 95%CI, 95% confidence interval
a Resolution of symptoms at 5 days was defined as the first day free of symptoms.
b Estimated difference is the difference in proportions shown for favipiravir compared to the control.
c Effect estimate is the odds ratio (2-sided 95%CI) from a logistic model.

Supplementary

Table 4 The residual symptoms on day five after favipiravir and standard treatment

Symptoms	Before Propensity Score Matching (Full Patient Cohort)				After Propensity Score Matching (Propensity Score Matched Patient Cohort)			
	Favipiravir (n = 888)	Standard treatment (n = 149)	Estimated difference (95% CI)[a]	P-value[b]	Favipiravir (n = 149)	Standard treatment (n = 149)	Estimated difference (95% CI)[a]	P-value[b]
	n	n			n	n		
Poor appetite	65 (7.3)	7 (4.7)	2.6 (-2.3 to 5.7)	0.244	15 (10.1)	7 (4.7)	5.4 (-0.8 to 11.4)	0.076
Anosmia	88 (9.9)	26 (17.4)	-7.5 (-14.7 to -1.9)	0.006	23 (15.4)	26 (17.4)	-2.0 (-10.4 to 6.5)	0.639
Conjunctivitis	6 (0.7)	0 (0.0)	0.7 (-1.9 to 1.5)	0.602	0 (0.0)	0 (0.0)	0.0 (-1.8 to 1.8)	NA
Diarrhea	38 (4.3)	5 (3.4)	0.9 (-3.5 to 3.4)	0.601	4 (2.7)	5 (3.4)	-0.7 (-4.9 to 3.6)	0.735
Runny nose	178 (20.0)	31 (20.8)	-0.8 (-8.4 to 5.6)	0.830	36 (24.2)	31 (20.8)	3.4 (-6.2 to 12.8)	0.488
Myalgia	127 (14.3)	28 (18.8)	-4.5 (-11.8 to 1.5)	0.155	21 (14.1)	28 (18.8)	-4.7 (-13.1 to 3.8)	0.274
Skin rash	30 (3.4)	7 (4.7)	-1.3 (-6.1 to 1.5)	0.422	4 (2.7)	7 (4.7)	-2.0 (-6.6 to 2.6)	0.357
Agusia	74 (8.3)	17 (11.4)	-3.1 (-9.4 to 1.5)	0.219	17 (11.4)	17 (11.4)	0.0 (-7.3 to 7.3)	1.000
Fatique	138 (15.5)	33 (22.1)	-6.6 (-14.3 to -0.2)	0.044	25 (16.8)	33 (22.1)	-5.4 (-14.3 to 3.7)	0.242
Sore throat	157 (17.7)	41 (27.5)	-9.8 (-17.9 to -2.8)	0.005	25 (16.8)	41 (27.5)	-10.7 (-19.9 to -1.3)	0.026
Dyspnea	238 (26.8)	47 (31.5)	-4.7 (-13.1 to 2.8)	0.230	42 (28.2)	47 (31.5)	-3.4 (-13.7 to 7.0)	0.527
Chest discomfort	40 (4.5)	6 (4.0)	0.5 (-4.2 to 3.2)	0.793	5 (3.4)	6 (4.0)	-0.7 (-5.2 to 3.9)	0.759
Cough	408 (45.9)	76 (51.0)	-5.1 (-13.6 to 3.5)	0.252	68 (45.6)	76 (51.0)	-5.4 (-16.6 to 6.0)	0.354

Abbreviations: n, number; 95%CI, 95% confidence interval

Data are presented as numbers (%).

a. An Estimated difference is the difference in proportions shown for favipiravir compared to the control.

b. P-value corresponds to the Chi-square test or Fisher’s exact test.

Supplementary

Table 5 Severity of adverse events in favipiravir and standard treatment

Adverse Events	Before Propensity Score Matching (Full Patient Cohort)							After Propensity Score Matching (Propensity Score Matched Patient Cohort)						
	Favipiravir (n = 888)			Standard treatment (n = 149)			p-value[a]	Favipiravir (n = 149)			Standard treatment (n = 149)			p-value
	Mild	Moderate	Severe	Mild	Moderate	Severe		Mild	Moderate	Severe	Mild	Moderate	Severe	
Nausea	56 (6.3)	10 (1.1)	5 (0.6)	14 (9.4)	2 (1.3)	4 (2.7)	0.032	11 (7.4)	3 (2.0)	2 (1.3)	14 (9.4)	2 (1.3)	4 (2.7)	0.744
Vomiting	16 (1.8)	10 (1.1)	1 (0.1)	7 (4.7)	2 (1.3)	2 (1.3)	0.012	4 (2.7)	3 (2.0)	0 (0.0)	7 (4.7)	2 (1.3)	2 (1.3)	0.460
Diarrhea	102 (11.5)	12 (1.4)	8 (0.9)	15 (10.1)	2 (1.3)	3 (2.0)	0.577	20 (13.4)	4 (2.7)	0 (0.0)	15 (10.1)	2 (1.3)	3 (2.0)	0.227
Eye discoloration	18 (2.0)	3 (0.3)	2 (0.2)	5 (3.4)	0 (0.0)	0 (0.0)	0.706	1 (0.7)	2 (1.3)	0 (0.0)	5 (3.4)	0 (0.0)	0 (0.0)	0.122
Jaundice	6 (0.7)	1 (0.1)	2 (0.2)	2 (1.3)	0 (0.0)	1 (0.7)	0.330	0 (0.0)	0 (0.0)	0 (0.0)	2 (1.3)	0 (0.0)	1 (0.7)	0.247
Arthralgia	42 (4.7)	9 (1.0)	1 (0.1)	6 (4.0)	3 (2.0)	0 (0.0)	0.588	11 (7.4)	0 (0.0)	0 (0.0)	6 (4.0)	3 (2.0)	0 (0.0)	0.111
Palpitation and sweating	58 (6.5)	7 (0.8)	2 (0.2)	10 (6.7)	3 (2.0)	1 (0.7)	0.235	9 (6.0)	1 (0.7)	0 (0.0)	10 (6.7)	3 (2.0)	1 (0.7)	0.615
Abdominal pain	7 (0.8)	6 (0.7)	1 (0.1)	2 (1.3)	0 (0.0)	0 (0.0)	0.660	0 (0.0)	0 (0.0)	0 (0.0)	2 (1.3)	0 (0.0)	0 (0.0)	0.498
Dizziness	67 (7.5)	14 (1.6)	3 (0.3)	14 (9.4)	2 (1.3)	3 (2.0)	0.093	13 (8.7)	0 (0.0)	0 (0.0)	14 (9.4)	2 (1.3)	3 (2.0)	0.177
Difficult breathing	27 (3.0)	8 (0.9)	2 (0.2)	7 (4.7)	2 (1.3)	0 (0.0)	0.554	2 (1.3)	2 (1.3)	0 (0.0)	7 (4.7)	2 (1.3)	0 (0.0)	0.247
Facial edema	6 (0.7)	1 (0.1)	1 (0.1)	1 (0.7)	0 (0.0)	0 (0.0)	1.000	2 (1.3)	0 (0.0)	0 (0.0)	1 (0.7)	0 (0.0)	0 (0.0)	1.000
Allergic rash	28 (3.2)	11 (1.2)	0 (0.0)	5 (3.4)	2 (1.3)	0 (0.0)	0.885	5 (3.4)	3 (2.0)	0 (0.0)	5 (3.4)	2 (1.3)	0 (0.0)	1.000

Abbreviations: n, number

Data are presented as numbers (%).

a P-value corresponds to the Chi-square test or Fisher's exact test.
